# Combined effect of nerve growth factor and brain-derived neurotrophic factor on neuronal differentiation of neural stem cells and the potential molecular mechanisms

**DOI:** 10.3892/mmr.2014.2393

**Published:** 2014-07-18

**Authors:** FEIFEI LIU, AIGUO XUAN, YAN CHEN, JUNDU ZHANG, LIPING XU, QIJIANG YAN, DAHONG LONG

**Affiliations:** 1 Department of Human Anatomy, Guangzhou Medical University, Guangzhou, Guangdong 510182, P.R. China; 2 The Second Affiliated Hospital, Guangzhou Medical University, Guangzhou, Guangdong 510260, P.R. China; 3 Kingmed Diagnostics College, Guangzhou Medical University, Guangzhou, Guangdong 510182, P.R. China; 4 Continuing Education College, Guangzhou Medical University, Guangzhou, Guangdong 510182, P.R. China

**Keywords:** neural stem cells, brain-derived neurotrophic factor, nerve growth factor, differentiation, basic helix-loop-helix

## Abstract

Neural stem cells (NSCs) are important pluripotent stem cells, which have potential applications in cell replacement therapy. Brain-derived neurotrophic factor (BDNF) and nerve growth factor (NGF) have been demonstrated to exert a marked impact on the proliferation and differentiation of NSCs. The effects of NGF, BDNF, and BDNF combined with NGF on NSC neuronal differentiation and the possible mechanisms for these effects were investigated in this study. An adherent monolayer culture was employed to obtain highly homogeneous NSCs. The cells were divided into four groups: Control, NGF, BDNF and combination (BDNF + NGF) groups. Neuron differentiation was examined using immunocytochemistry and phospho-extracellular signal-regulated kinase (p-ERK) levels were analyzed using western blotting. Reverse transcription polymerase chain reaction was used to measure the mRNA expression levels of the HES1, HES5, MASH1, NGN1 and NeuroD transcription factors at different time intervals following neurotrophin-induced differentiation. NGF and BDNF were observed to induce NSC neuronal differentiation, and β-tubulin III-positive cells and p-ERK expression levels were highest in the NGF + BDNF combination group at all time points. The proportion of β-tubulin III-positive neurons in each group was associated with the expression levels of MASH1, NGN1 and NeuroD in the group. In conclusion, BDNF combined with NGF significantly improved NSC neuronal differentiation, which may provide support for the practical application of NSCs in neurodegenerative diseases.

## Introduction

Neural stem cells (NSCs) are generated throughout adult life via the process of neurogenesis (1). NSCs replace lost or damaged neurons and are capable of differentiating into excitatory granule neurons, which are involved in learning and memory (2,3). Various roles of NSCs in diseases have been elucidated by several studies (2,4,5) and NSCs may have promising applications in the treatment of human neurological diseases, such as Alzheimer’s disease and amyotrophic lateral sclerosis (6).

Brain-derived neurotrophic factor (BDNF) is fundamental for learning and long-term memory in the central nervous system (7). Nerve growth factor (NGF) is critical for the survival and maintenance of sympathetic and sensory neurons, and induces axonal growth. These neurotrophin (NT) factors, BDNF and NGF, are critical for the maintenance and differentiation of developing neurons (8). In a recent study, Kumamaru *et al* (9) observed neurotrophin receptor expression on the cell membranes of NSCs and identified the activation of TrkB by BDNF, a process that supports neuron survival and promotes differentiation of developing neurons. NGF activation of TrkA is also critical in inducing cellular survival and differentiation (10). Furthermore, the activation of TrkA and TrkB by BDNF and NGF activates Ras, and promotes the activation of extracellular signal-regulated kinase (ERK) (9,10).

A basic helix-loop-helix (bHLH) is a protein structural motif that characterizes a family of transcription factors (TFs) (11). TF expression is important in the proliferation, growth and differentiation processes of NSCs (12): HES1 and HES5 maintain the number and status of undifferentiated NSCs and neural progenitor cells *in vivo* and *in vitro* (13,14); MASH1 expression in NSCs induces morphological differentiation and expression of neuronal markers (15,16); and NGN1 and NeuroD have been demonstrated to be important in neural differentiation (17). MASH1, NGN1 and NeuroD expression has been found to be induced by BDNF and/or NGF in NSCs (18,19).

Previous studies have documented the differences among NGF, BDNF and combined treatments on NSCs (18,20). However, the underlying mechanisms remain unclear. Another previous study confirmed that NGF 50 μg/l or BDNF 40 μg/l treatment was capable in giving rise to more neurons three days after NT administration (21). In the present study, the differential effects of individual or combined NGF and BDNF treatments on NSCs were investigated, along with the possible mechanisms.

## Materials and methods

### Isolation and culture of NSCs from rat embryo cerebra (embryonic day 14–16)

Pregnant Sprague Dawley (SD) rats were provided by the Guangdong Medical Laboratory Animal Center (Guangzhou, China). Rat mothers were euthanized with overdose of anesthetic and the whole body was disinfected with 75% vol/vol ethanol. Embryonic rats were removed in 75% vol/vol ethanol, after 1–2 min embryos were transferred to cold Dulbecco’s modified Eagle’s medium (DMEM)/F12 (Gibco-BRL Carlsbad, CA, USA) in sterile working conditions and decapitated. All animal experiments were performed according to protocols approved by the ethics committee of the Guangzhou Medical University, Guangzhou, China). Rat embryonic cerebra were separated and triturated to single cells in sterile working conditions. The cells were centrifuged at 200 × g for 5 min at room temperature, then suspended in fresh proliferation medium, which consisted of 98% DMEM/F12, 2% B27 (Gibco-BRL), 20 μg/l basic fibroblast growth factor (bFGF, Shanghai PrimeGene Bio-Tech Co., Ltd., Shanghai, China) and epidermal growth factor (EGF, Shanghai PrimeGene Bio-Tech Co., Ltd.), to a final cell density of 5×10^5^ cells/ml. The culture medium was changed every 48 h. The secondary neurospheres were gathered and digested by Accutase (Sigma-Aldrich, St. Louis, MO, USA) to form single cells. The cells were then plated onto poly-D-lysine- (200 mg/l, Sigma-Aldrich) coated 24- or 6-well plates at a final cell density of 5×10^4^ cells/cm^2^ and cultured as an adherent monolayer. Every 48 h, 50% of the medium was changed with fresh aliquots.

### Inducing NSC differentiation

While the culture grew to 75–80% confluence, the cells were cultured with differentiation medium, which consisted of 24.5% DMEM/F12, 74% Neurobasal (Gibco-BRL), 1% B27 and 0.5% N_2_ (Gibco-BRL). The cells were divided into four groups: Control, NGF 50 μg/l, BDNF 40 μg/l and BDNF combined with NGF. The medium used to induce the cells contained either BDNF or NGF, a combination of BDNF and NGF, or neither NT in order to serve as the negative control. The differences among the control, NGF, BDNF and combination groups were compared on days 1, 3, 7 and 14 after induction.

### Immunocytochemistry/immunofluorescence

The medium was removed and the cells were fixed with 4% paraformaldehyde for 20 min. The cells were blocked with 10% bovine serum albumin and 0.3% Triton-X 100 for 30 min at room temperature. The cells were incubated with primary antibodies [rabbit monoclonal anti-β-tubulin III antibody (1:300; Sigma-Aldrich) or mouse monoclonal anti-Nestin antibody (1:200; Abcam, Cambridge, UK)] for 36 h at 4°C. The cells were then incubated with secondary antibodies [tetramethylrhodamine isothiocyanate-conjugated goat polyclonal secondary antibody to rabbit IgG (1:300; Abcam), fluorescein isothiocyanate (FITC)-conjugated goat anti-rabbit IgG (1:100; Boiss, Inc., Beijing, China), or FITC-conjugated goat anti-mouse IgG (1:100; Boiss, Inc.)] for 3 h at room temperature with agitation. The cells were then incubated with DAPI (100 μg/l) for 10 min.

### Western blotting (WB)

Mitogen-activated protein kinase kinase (MEK) is upstream of ERK. At 30 min after treatment with the MEK inhibitor PD98059 (10 μM; Cell Signaling Technology, Inc., Danvers, MA, USA), the level of phospho-ERK (p-ERK) was detected by WB.

Samples of 10 μg total protein were separated in SDS-PAGE gel. The proteins were then transferred onto a polyvinylidene fluoride (PVDF) membrane. The PVDF membrane was blocked for 2 h at room temperature and then incubated with primary antibodies [rabbit anti-phospho-p44/42 MAPK (ERK1/2; Thr202/Tyr204; 1:1,000; Cell Signaling Technology, Inc.), rabbit anti-p44/42 MAPK (ERK1/2; 1:1,000; Cell Signaling Technology, Inc.) or rabbit anti-GAPDH antibody (1:5,000; Abcam)] overnight with gentle agitation at 4°C. The following day, the membrane was incubated with secondary antibodies [horseradish peroxidase-conjugated goat anti-rabbit IgG, (1:5,000; CoWin Biotech Co., Ltd., Beijing, China)] for 3 h at room temperature. Protein detection was performed using enhanced chemiluminescence.

### Reverse transcription polymerase chain reaction (RT-PCR)

A total of 2 μg total RNA was reverse transcribed to cDNA using a PrimeScript^TM^ RT reagent kit (Takara Bio, Inc., Shiga, Japan); 2 μl cDNA was then detected by RT-PCR in a 20 μl reaction mixture containing 0.2 μM of each of the paired primers using a SYBR Premix Ex Taq^TM^ (Takara Bio, Inc.). The specific primer pairs are shown in Table I.

Prior to commencing the amplification, the samples were preheated at 95°C for 30 sec. The RT-PCR conditions consisted of 40 cycles of 5 sec at 95°C and 34 sec at 60°C. The dissociation stage consisted of 15 sec at 95°C, 1 min at 60°C and 15 sec at 95°C. The gene expression levels were normalized against endogenous β-actin and NSCs served as a reference sample.

### Statistical analysis

All statistical analyses were computed using SPSS 17.0 software (SPSS, Inc., Chicago, IL, USA). Comparisons among all groups were analyzed by one-way analysis of variance, and F-test and Student’s t-test were used for comparisons between two groups. P<0.05 was considered to indicate a statistically significant difference.

## Results

### Combined BDNF and NGF treatment induces more neurons than BDNF or NGF alone

**Figure 1:**
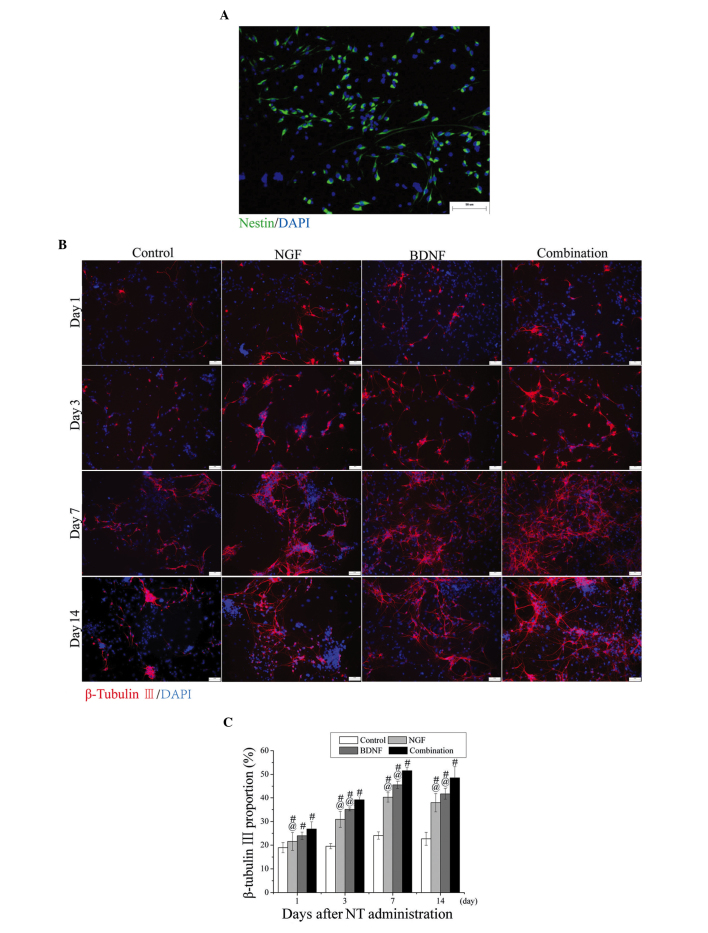
Expression levels of β-tubulin III in different groups. (A) Neural stem cells (NSCs) cultured as a monolayer were detected by anti-Nestin antibody at two days after being plated. (B) NSC differentiation was induced with nerve growth factor (NGF; 50 μg/l), brain-derived neurotrophic factor (BDNF; 40 μg/l) or a combination of BDNF + NGF. β-tubulin III positive cells in different groups were stained at 1, 3, 7 and 14 days after neurotrophin-induced differentiation. (C) The proportions of β-tubulin III-positive cells in different groups were analyzed by SPSS software. ^#^P<0.05 vs. control; ^@^P<0.05 vs. combination treatment; n=3, bar=50 μm.

NSC growth was evaluated by anti-Nestin antibody two days after the cells were plated as an adherent monolayer. The results revealed that the proportion of Nestin-positive cells was 93.17±3.06% (Fig. 1A). The proportions of β-tubulin III-positive cells were counted and compared among the four groups. The percentage of β-tubulin III-positive neurons was significantly lowest in the control group at all time intervals (P<0.05). The differences between the NGF and combination groups were statistically significant at all time points (P<0.05). Although no significant difference between the BDNF and the combination groups was detected on day 1 after administration, statistically significant differences were identified on days 3, 7 and 14 after administration (P<0.05; Fig. 1B and C). These results demonstrate that the combination treatment induced more neurons than BDNF or NGF alone.

### p-ERK activation may be involved in NSC neural induction by NGF, BDNF or combination treatment

**Figure 2:**
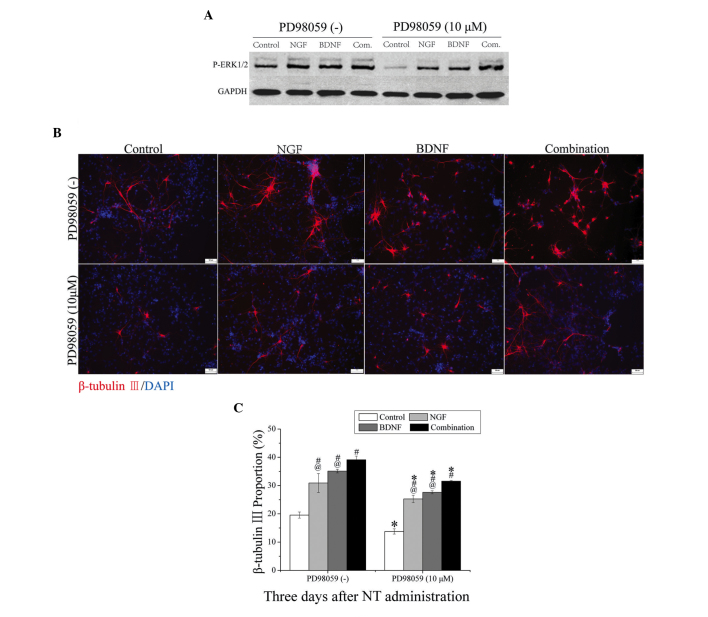
Activation of extracellular signal-regulated kinase (ERK)1/2 was involved in neurotrophin-induced differentiation. (A) Phospho-ERK and GAPDH expression levels were analyzed by western blotting (Com. = Combination group, BDNF + NGF). (B and C) Neural stem cells were treated with or without PD98059 (30 min), followed by administration of nerve growth factor (NGF), brain-derived neurotrophic factor (BDNF) or BDNF + NGF for three days. The proportions of β-tubulin III-positive cells in different groups were detected by immunofluorescence. ^*^P<0.05 vs. corresponding PD98059 (−); ^#^P<0.05 vs. control under the same PD98059 condition; ^@^P<0.05 vs. combination under the same PD98059 condition; n=3, bar=50 μm.

At 30 min after treatment with the MEK inhibitor PD98059, the level of p-ERK was significantly reduced compared with the p-ERK level following control treatment. BDNF and NGF treatments were shown to activate ERK, as the p-ERK expression levels were significantly higher in the NGF, BDNF and combination groups, in comparison with the control group, although this was partly inhibited by PD98059 (Fig. 2A and B). To further determine whether inhibition of the MAPK/ERK signaling pathway affected the cell phenotype, at day 3 after NT administration, the immunocytochemical results regarding β-tubulin III further confirmed the WB results. The results showed that PD98059 significantly inhibited neuronal differentiation in the NGF, BDNF and combination groups (P<0.05; Fig. 2C and D).

### Changes in the expression levels of bHLH transcription factors are induced by NTs

**Figure 3:**
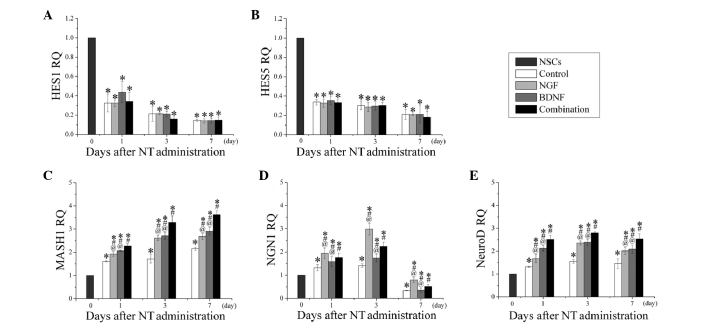
Changes in the mRNA expression levels of basic helix-loop-helix transcription factors. β-actin served as an endogenous control gene. The neural stem cell (NSC) group served as a reference sample. (A–E) Relative quantity (RQ) levels of HES1, HES5, MASH1, NGN1 and NeuroD in different groups at days 1, 3 and 7 after neurotrophin-induced differentiation. ^*^P<0.05 vs. NSCs; ^#^P<0.05 vs. control; ^@^P<0.05 vs. combination treatment; n=3.

The RT-PCR results demonstrated that the relative quantity (RQ) of HES1 was markedly reduced following the withdrawal of EGF and bFGF. No significant difference was detected among the control, NGF, BDNF and combination groups at any time point (Fig. 3A). The HES5 RQ exhibited a similar variation trend to HES1 (Fig. 3B). The expression levels of MASH1 were significantly enhanced in the differentiation-induced groups as compared with the control groups (P<0.05). The MASH1 RQ in the combination group was significantly increased in comparison with the NGF (P<0.05, Fig. 3C) or BDNF groups (P<0.05, Fig. 3C) at all time points. The NGN1 expression levels were significantly enhanced compared with the control and NSC groups on days 1 and 3 after NT-induced differentiation (P<0.05), but were significantly reduced on day 7, compared with the NSC group (P<0.05). The NGN1 expression levels in the combination group were significantly higher than those of the BDNF group at all time points (P<0.05, Fig. 3D). Notably, compared with the combination group, higher NGN1 expression levels were observed in the NGF group at all time points (P<0.05, Fig. 3D). NeuroD expression levels were significantly enhanced at all time points following NT-induced differentiation, compared with the control group (P<0.05), and the combination group induced a higher quantity than in the NGF (P=0.001, Fig. 3E) or BDNF groups (P=0.005, Fig. 3E).

## Discussion

An adherent monolayer culture was employed to obtain highly homogeneous NSCs. The cells were uniformly exposed to culture medium that supports symmetrical cell division and equal administration of NTs. Monitoring the morphology and survival of undifferentiated and differentiated cells is important in order to select a comparably vital culture for experiments (22). This method was useful to transform neurospheres into a monolayer culture, which may occasionally yield a purer population of Nestin-positive cells (23). A previous study revealed that NSCs from the embryonic rat brain in an adherent monolayer culture were capable of developing into neurons, which was determined by knowledge of the cellular responses to specific growth factors. The selection of NGF or BDNF dosage depends on the research aim and experimental method. In the present study, the data confirmed that NGF 50 μg/l and BDNF 40 μg/l were able to give rise to the maximum number of neurons at day 3 of treatment, as the NSCs were induced to differentiate. Nestin-positive cells existed until day 3 after NT administration (data not shown), which suggests that NSC differentiation may be asynchronous. Notably, the proportion of β-tubulin III-positive cells peaked at day 7 but was reduced at day 14. Cell death may be involved at late differentiation stages, since cell fragments adhered to the wall surface were detected at day 10 after differentiation-induction.

The present study revealed that NGF or BDNF treatment increased the proportion of differentiated neurons, and BDNF combined with NGF treatment induced the development of more neurons than BDNF alone. Several recent studies have demonstrated that NTs markedly enhance neuronal differentiation of NSCs (24,25). Although increased doses of NGF or BDNF did not improve the neuronal differentiation proportion, BDNF combined with NGF did enhance this proportion. NGF (50 μg/l) may saturate TrkA receptors, thus no extra receptors are able to bind the extra NGF sites, and an identical scenario occurs with BDNF-TrkB. However, NGF-TrkA and BDNF-TrkB binding jointly activates the intrinsic receptor tyrosine kinase, enhancing the ERK phosphorylation level and the associated signaling pathways and therefore promoting cell differentiation (26). There is no question that the BDNF and NGF combination was an improved induction treatment for neurons in comparison with either BDNF or NGF treatment alone.

BDNF has been demonstrated to markedly increase the level of p-ERK through binding to TrkB (27), suggesting that BDNF may induce changes in the ERK signaling pathway. In the present study, BDNF combined with NGF produced a greater increase in p-ERK levels than BDNF alone, which indicates that, although TrkA shares the same downstream with TrkB, the two kinases do not impact each other. Usage of the MEK inhibitor PD98059 further confirmed the result. BDNF and NGF compete with PD98059 to affect the signaling pathway. Even following previous incubation with PD98059, p-ERK levels in NSCs may also be increased by administration of NGF or BDNF alone, or the combination of the two treatments. Cytological results revealed that the proportions of β-tubulin III-positive cells in different groups exhibited the same pattern as the p-ERK levels in the groups, which indicates that ERK promoted neuronal differentiation.

Combinatorial regulation of TFs by NTs is also important in determining the neuronal gene expression levels. HES1 and HES5 are highly expressed in NSCs, which are known as repressor-type differentiation genes. Subsequent to withdrawing EGF and bFGF, the RQs of HES1 and HES5 were significantly reduced. HES1 functionally antagonizes MASH1 by repressing gene expression and inhibiting protein activity. Administration of NGF or BDNF markedly increased the expression levels of MASH1 compared with the control group, and BDNF combined with NGF exerted a significant increase in MASH1 expression levels at all time intervals when compared with either treatment alone. BDNF was found to induce the Wnt/β-catenin signaling pathway (24) and MASH1 has been reported as a downstream molecule of the Wnt/β-catenin signaling pathway (11). The trend in MASH1 expression levels was associated with the proportion of β-tubulin III-positive cells in different groups at all time points. This indicates that MASH1 was expressed in immature and mature neurons, and that MASH1 promoted neural differentiation and the maintenance of the neuronal phenotype. NGN1 expression levels were enhanced on days 1 and 3 after NT-induced differentiation, but were reduced on day 7. NGN1 is expressed in neural precursors and neurons (17), and induces neuronal differentiation of neural precursors (19). The findings of the present study were in accordance with these results. The NGN1 expression levels were highest in the NGF group and lowest in the BDNF group. Since NGN1 regulates specification of neuron type (28), this indicates that NGF and BDNF may induce different neuron subtypes (29). In addition to the combined effect, BDNF and NGF exerted a competitive effect on neuronal precursors in the combination group. NeuroD drives neural differentiation of precursor cells (30). In the results of the present study, NeuroD expression levels were increased the most in the combination group, and were enhanced in immature and mature neurons following the beginning of differentiation towards a neural phenotype.

In conclusion, the roles of NGF, BDNF and the BDNF and NGF combination in inducing the neuronal differentiation of NSCs were investigated in the present study. The combination of BDNF and NGF induced more neurons than NGF or BDNF alone. Furthermore, p-ERK activation and alterations in the expression levels of bHLH transcription factor mRNAs were involved in neural induction of NSCs by NGF, BDNF or the combination of the two. The results demonstrated that BDNF combined with NGF exerts a combined effect on NSC neuronal differentiation.

## Figures and Tables

**Table I:** qPCR primers used to detect gene expression levels in the NSC developmental process.

Gene	Primer sequence
β-actin	F: 5′-GAGACCTTCAACACCCCAGC-3′ R: 5′-ATGTCACGCACGATTTCCC-3′
HES1	F: 5′-TAACGCAGTGTCGCCTTCC-3′ R: 5′-AGAGGTGGGCTAGGGAGTTTATG-3′
HES5	F: 5′-AGCCGGTGGTGGAGAAGAT-3′ R: 5′-AGTTTGGAGTTGGGCTGGTG-3′
MASH1	F: 5′-AGGCCCTACTGGGAATGGA-3′ R: 5′-CCCTGTTGCTGAGAACATTGA-3′
NGN1	F: 5′-GACACCCTGCTTCATCCCGTA-3′ R: 5′-TCTTTAAAGCTCCCAGCATCCAC-3′
MASH1	F: 5′-TGCACCAGCCCTTCCTTT-3′ R: 5′-CGGTGGATGGTTCGTGTTT-3′

qPCR reactions were conducted in 7500 PCR Applied Biosystems. qPCR, quantitative transcription polymerase chain reaction; NSC, neural stem cell; F, forward; R, reverse.
